# Setting a research agenda for the use of extended reality in healthcare simulation: an Utstein style meeting

**DOI:** 10.1186/s41077-026-00409-y

**Published:** 2026-03-03

**Authors:** Barry Issenberg, Doris Ostergaard, Francisco Matos, Pier Luigi Ingrassia, Kirsty Freeman, Lars Konge, Carla Sa-Couto, Gabriel Reedy, The Utstein XR in Simulation Expert Group, Asmita Acharya, Joana Berger-Estilita, Kristen Brown, Lennox Huang, M. Emin Aksoy, Marc Lazarovici, May Sissel Vadla, Myriam Alami Younsi, Olivia Dow, Peter Dieckmann, Samia Barbar, Scott Crawford, Serena Ricci, Todd P. Chang, Tore Laerdal, Vicki LeBlanc

**Affiliations:** 1 University of Miami Gordon Center for Simulation and Innovation in Medical Education, 1120 Northwest 14th Street, Miami, FL 33136 USA; 2 Copenhagen Academy for Medical Education and Simulation, Ryesgade 53B, Copenhagen, 2100 Denmark; 3 University of Coimbra, Paco das Escolas, Coimbra, 3000-370 Portugal; 4 Centro Di Simulazione (CeSi), Via Ronchetto 14, Lugano, Ticino 6900 Switzerland; 5 The University of Western Australia, M Block, QEII Medical Centre, Caladenia Cres (Entrance Off Hampden Road) Nedlands WA: MBDP 709U, 35 Stirling Highway, Perth, WA 6009 Australia; 6 RISE-Health, Community Medicine, Information and Decision Sciences Department (MEDCIDS), Faculty of Medicine, University of Porto (FMUP), Porto, Portugal; 7 Kings College, Strand, London, WC2R 2LS UK; 8 Department of Clinical Medicine, University of Copenhagen, Copenhagen, Denmark

**Keywords:** Extended Reality (XR), Healthcare simulation, Simulation-based education, Utstein style meeting, Virtual Reality (VR), Augmented Reality (AR), Mixed Reality (MR), Research agenda

## Abstract

**Background:**

Extended reality (XR), encompassing virtual, augmented, and mixed reality, is increasingly integrated into healthcare simulation-based education and training. While XR offers immersive, scalable, and potentially cost-effective learning environments, evidence of its educational effectiveness and implementation remains fragmented. To address this gap, an Utstein-style consensus meeting was convened to establish an international research agenda that advances the evidence-based integration of XR in healthcare education.

**Methods:**

The meeting, held in Copenhagen, Denmark, in November 2024, brought together 24 international experts representing diverse disciplines, geographic regions, and simulation expertise. A Delphi-informed pre-meeting survey, based on a targeted review of XR-focused literature, identified key topics across five initial domains. Using an implementation science–informed approach guided by the NASSS (Non-adoption, Abandonment, Scale-up, Spread, and Sustainability) framework, participants engaged in structured breakout and plenary discussions to explore barriers, facilitators, and research priorities related to XR adoption and sustainability.

**Results:**

Through consensus deliberation, the original five domains were refined into three overarching themes: (1) uses and adoption of XR, (2) barriers to XR adoption and implementation, and (3) facilitators of XR implementation and sustainability. Each theme comprised multiple subthemes addressing critical issues such as technological definitions, economic and infrastructure needs, human resource and faculty development, usability, educational quality, customization, and ethical considerations. Research questions within each subtheme were classified as descriptive, justificatory, or clarificatory to guide future study design and methodological rigor.

**Conclusions:**

This Utstein Style meeting represents the first international, consensus-based effort to develop a structured research agenda for XR in healthcare simulation. Informed by implementation science, particularly the NASSS framework, the resulting agenda provides a roadmap for future research, policy, and practice. It emphasizes faculty readiness, institutional investment, and data-driven evaluation as prerequisites for sustainable integration. By addressing barriers and leveraging facilitators, the healthcare simulation community can advance the responsible adoption of XR technologies, enhancing competency, equity, and innovation in health professions education worldwide.

**Supplementary Information:**

The online version contains supplementary material available at 10.1186/s41077-026-00409-y.

## What this paper adds

- This Utstein Style Meeting established the first international research agenda focused on the use of extended reality (XR) in healthcare simulation.
- The consensus process identified priority areas spanning technology, economics, implementation, educational impact, and ethics to guide evidence-based adoption of XR.
- The resulting research questions provide a framework for advancing the science of XR in health professions education, supporting its responsible and sustainable integration.
- These findings highlight the need for coordinated, multidisciplinary collaboration to ensure XR innovations enhance learning outcomes, promote equity, and strengthen the global simulation community.

## Background and introduction

Simulation-Based Education and Training (SBET) is increasingly recognized for its numerous benefits over traditional training modalities. It provides an immersive, contextual environment where both individuals and teams can develop, hone, and evaluate technical, cognitive, and social competencies. This methodological shift towards a more interactive and reflective learning framework allows for the acquisition of skills in a controlled, ethical, and safe setting. In an effort to provide guidance on best practices and research directions, there have been different approaches to guide the healthcare simulation community. These include Utstein style meetings [1, 2] and simulation society research summits [3–5].

In a collaborative effort, the Society for Simulation in Europe (SESAM) and the Society for Simulation in Healthcare (SSH) facilitated the achievement of a Global Consensus Statement on simulation-based practice [6]. This unified declaration, crafted by representatives from 50 national and international simulation societies and networks distributed across 67 countries, addresses the current challenges within healthcare systems and the various impacts on healthcare professionals, proposing SBET as a potential solution to some of these issues. One of the recommended strategies was to leverage virtual and technology-enhanced forms of simulation to expand accessibility across professions and practice settings. While immersive simulations have long been integral in training healthcare professionals, effectively enhancing performance and outcomes, they are often associated with considerable costs. Concurrently, technological advancements have led to the emergence of innovative immersive methods that incorporate virtual reality (VR), augmented reality (AR), and mixed reality (MR). Collectively referred to as extended reality (XR), these technologies promise to revolutionize the training landscape for healthcare professionals by expanding the available educational tools offering diverse and potentially more cost-effective training solutions. For the purpose of this project, the authors used definitions of these technologies contained in the Healthcare Simulation Dictionary v3 [7]. The authors also recognized that the distinctions between VR, AR, and MR are often not clearly delineated in practice and that a blend of these technologies are often used in healthcare simulation (Table 1).

**Table 1:** Definition of extended reality technologies and examples of their use in healthcare simulation

Term	Definition*	Example in Healthcare Simulation
AR (Augmented Reality)	A technology that overlays digital information on objects or places in the real world for the purpose of enhancing the user experience”	AR display headsets can be used in clinical training to display anatomical images directly in the field of view on top of a physical simulator to provide guidance about important landmarks for procedures
MR (Mixed Reality)	A technology that seamlessly blends the user’s real-world environment with digitally-created content, where both environments can coexist and interact with each other	MR technology is used to overlay dynamic, interactive 3D visualizations of patient physiology, such as real-time cardiac output and oxygen saturation, onto a manikin, allowing learners to engage simultaneously with both the physical simulator and holographic clinical data for enhanced decision-making and situational awareness
VR (Virtual Reality)	A computer-generated, three-dimensional virtual environment that users can interact with, typically accessed via a computer that is capable of projecting 3D information via a display, which can be isolated screens or a wearable display Non-immersive VR utilizes a combination of screens surrounding the user to present virtual information Immersive VR refers to using a wearable display, … to track a user’s movement and present the VR information based on the position of users, which allows them to experience 360 degrees of the virtual environment	Non-immersive—screen-based scenarios that user interacts with avatars in a virtual environment to learn about the navigating the space and operating the equipment in an operating room environment Immersive—A VR-based simulation platform such as Trauma Resuscitation using In-Situ Simulation Team Training immerses medical trainees in a high-stakes trauma bay environment. Learners wear VR headsets to practice the assessment and management of multi-system trauma patients

^*^From Healthcare Simulation Dictionary V3, 2025

Despite their increasing use, the impact of these XR technologies on actual learning remains underexplored. Current research primarily focuses on participant satisfaction, with fewer studies assessing learning outcomes or offering specific guidance on selecting the appropriate technological method for particular educational goals [8, 9]. One comprehensive integrative review concluded that while XR technologies are often as effective as traditional education methods and may offer scalability and cost benefits, many studies were exploratory and varied in design. They concluded that “further research to compare different variations of XR technologies and [identify] best applications in medical education and training are required to advance the field.” [8]​ In another review of virtual reality head-mounted displays in medical education, the authors found strong learner preference and promising outcomes for surgical training, but inconclusive evidence for better learning in other domains, such as anatomy, communication skills, and clinical decision making [10]. Finally, a recent systematic review of XR in resuscitation training found limited and inconsistent evidence of its effectiveness compared to traditional methods, with most studies favoring other interventions or showing no significant difference [11]. These findings highlight the need for more rigorous research to evaluate how and when XR can most effectively enhance learning outcomes in healthcare simulation [12].

Some XR developments are the result of close collaborations with healthcare professionals and educationalists, aimed at achieving learning objectives that are challenging with traditional methods [13]. However, much of XR development, innovations, and utility comes from the games and entertainment industry that is minimally related to healthcare SBET [14]. This is a challenge for the healthcare XR SBET space that is not relevant with mannequin-based simulators, task trainers and standardized patients whose methodology and purpose were specifically crafted for healthcare simulation. Moreover, there is a pressing need for better understanding of how to train faculty and staff to effectively plan and implement educational interventions using these novel XR technologies within existing curricula [15].

The Utstein style meetings have historically played a crucial role in shaping the research agenda for several aspects of healthcare simulation. These meetings have led to significant publications that have not only facilitated the development of shared research agendas but also highlighted critical topics for enhancing patient safety and detailed descriptions of national programs [1, 4, 5, 16]. The accumulated insights from these meetings continue to inform and guide the evolving practices within healthcare SBET. In addition, simulation societies have arranged several research meetings and published the outcomes to share the current state of the science in healthcare SBET and to collaboratively map future research priorities [6–8]. The co-organizers of this project have extensive experience leading similar initiatives within their respective simulation societies and have previously applied the Utstein style approach to simulation research agenda–setting efforts [1–3].

The primary objective of this recent Utstein style meeting was to forge consensus on several key aspects of emerging XR technologies in healthcare simulation. To structure our analysis of extended reality (XR) use in healthcare simulation, we adopted an implementation-science–informed approach, drawing on the Non-adoption, Abandonment, Scale-up, Spread, and Sustainability (NASSS) framework proposed by Greenhalgh et al. This framework was developed to explain why some health technologies are successfully adopted and sustained while others are not, emphasizing that “poor uptake of technological innovations is often explained in terms of barriers and facilitators.” [17] Guided by NASSS, our study sought to: (1) characterize current uses and adoption of XR in health professions education; (2) identify barriers to its adoption and implementation; and (3) determine key facilitators that may support broader implementation and sustainability. By grounding our inquiry in an established implementation framework, we aimed to move beyond descriptive accounts of XR use toward a structured understanding of the factors influencing its effective translation into educational practice.

## Process to develop an extended reality healthcare simulation research agenda—Utstein style meeting

### Selection of participants

Participants were selected to ensure international, multidisciplinary, and multiprofessional representation among experts in simulation research and extended reality (XR) technology. The organizers invited individuals with a strong research record, demonstrated experience with XR, and active involvement in collaborative projects. The group reflected diversity across professions (e.g., nursing, medicine, psychology) and geographic regions (Africa, Europe, North America, and South America). Following the traditional format of Utstein style meetings, which typically include approximately 24 participants to balance representativeness and effectiveness, 26 experts were invited in addition to the four organizers Two of these were not able to attend the in person meeting—Table 2).

**Table 2:** Summary of Participants in the Utstein Style Meeting

Participant	Country	Institution
Asmita Acharya, MD	Norway	Laerdal Global Health
Barry Issenberg, MD	United States	University of Miami Gordon Center for Simulation and Innovation
Carla Sá-Couto, PhD	Portugal	University of Porto
Doris Østergaard, MD	Denmark	Copenhagen Academy for Medical Education & Simulation
Francisco Maio Matos, MD, PhD	Portugal	University of Coimbra
Gabrial Reedy, PhD	United Kingdom	Kings College, London
Joana Berger-Estilita, MD, PhD	Switzerland	University of Bern
Kirsty Freeman, PhD	Australia	The University of Western Australia
Kristen Brown, DNP, CRNP	United States	Johns Hopkins Univ School of Nursing
Lars Konge, MD, PhD	Denmark	Copenhagen Academy for Medical Education and Simulation
Lennox Huang, MD, MBA	Canada	The Hospital for Sick Children, Toronto
M. Emin Aksoy, MD, PhD	Türkiye	Acıbadem University
Marc Lazarovici, MD	Germany	Ludwig-Maximilians-University
May Sissel Vadla, MD	Norway	SAFER, Stavanger
Myriam Alami Younsi, MD	Morocco	Moroccan Society for Simulation in Healthcare
Olivia Dow, MD	United Kingdom	Royal Surrey County Hospital, Guildford, UK
Peter Dieckmann, PhD	Denmark	Copenhagen Academy for Medical Education and Simulation; Department of Quality and Health Technology, University of Stavanger, Stavanger, Norway; Department of Public Health, Copenhagen University, Copenhagen, Denmark
Pier Luigi Ingrassia, MD, PhD	Italy	Centro di Simulazione (CeSi), Lugano, Ticino, Switzerland
Samia Barbar, MD, PhD^c^	Brazil	University of Miami Gordon Center for Simulation and Innovation
Scott Crawford, MD	United States	Texas Tech Univ Health Sciences Center at El Paso
Serena Ricci, PhD	Italy	University of Genoa
Todd P Chang, MD, MAcM	United States	Children’s Hospital Los Angeles Las Madrinas Simulation Center/University of Southern California Keck School of Medicine
Tore Laerdal	Norway	Executive Director, Laerdal Foundation
Vicki LeBlanc, PhD	Canada	University of Ottawa

### Preparation of the Utstein style meeting

The organizing team, the main authors of this article, planned the meeting to leverage participants’ collective expertise through an inductive, consensus-building process. To inform discussions, the organizers conducted a preliminary literature review and pre-meeting Delphi-style survey. A scoping search identified review articles published within the past decade on the use and effectiveness of XR (virtual, augmented, and mixed reality) in healthcare education and training. The search was supported by *Elicit: The AI Research Assistant* (Oakland, CA), which helped categorize key studies by design, methods, findings, and limitations (see Appendix 1 for the 21 identified references). Based on the literature review, an initial Delphi survey was developed to identify and prioritize topics across five domains: (1) uses of XR to address educational gaps; (2) barriers to XR implementation; (3) facilitators of XR use; (4) faculty development needs; and (5) research priorities.

Following review of the survey results and preliminary discussions among the organizers, these five domains were refined into three core focus areas for the Utstein style meeting:

1. Current uses and adoption of XR in healthcare education and training.
2. Barriers to XR adoption and implementation.
3. Facilitators that support implementation and sustainability.

Research questions were framed around each of these three areas to guide consensus discussions. The domain focused on faculty development was determined to be outside the immediate scope of this meeting and will be addressed separately in a future initiative.

The survey achieved an 88% response rate (23 of 26 invitees). Items rated as highly important (scores of 4 or 5 on a 5-point Likert scale) by at least 75% of respondents were prioritized for consensus-building discussions (Appendix 3). Free-text comments were analyzed and grouped with related items to enrich discussion and contextualize priorities during the meeting.

### Process of the Utstein style meeting

The Utstein style meeting was held in November 2024 in Copenhagen, Denmark, and began with an informal evening gathering to foster an open and collaborative working environment (agenda provided in Appendix 4).

#### Day 1— setting the stage and discussion rounds

The meeting opened with an overview of the Utstein framework and its relevance to advancing simulation-based practice, as outlined in the Global Consensus Statement on Simulation-Based Practice in Healthcare [6]. Plenary presentations introduced shared definitions of extended reality (XR) and summarized findings from the pre-meeting Delphi survey to orient participants to key themes. Participants were assigned to multidisciplinary breakout groups co-facilitated by the organizing authors. Each group discussed one of the three focus areas. Discussions were documented electronically using collaborative tools (e.g., Microsoft Copilot, Redding, WA), and synthesized summaries were presented in plenary sessions for clarification and collective reflection. This structure, brief plenary orientation followed by focused small-group discussion and synthesis, was repeated for each topic area to promote cross-pollination of ideas and consensus building.

#### Day 2 – developing the research agenda

The second day centered on generating and prioritizing research questions derived from the themes discussed on Day 1. The morning began with a summary of synthesized insights and a brief introduction to a structured framework for formulating research questions. Participants again worked in small, multidisciplinary groups assigned to one of the three domains: (1) uses and adoption of XR, (2) barriers to adoption and implementation, and (3) facilitators of implementation and sustainability. Each group identified knowledge gaps, brainstormed corresponding research questions, and prioritized them based on relevance, feasibility, and potential impact.

In the afternoon, groups refined their proposed research questions based on plenary feedback. This iterative process involved consolidating overlapping ideas, aligning topics with broader educational and implementation priorities, and finalizing a prioritized set of research questions for each domain. The resulting research agenda represents a consensus view of the key directions needed to advance XR integration in healthcare simulation.

## Research agenda and proposed research questions

Deliberations during the Utstein meeting produced a series of prioritized research questions intended to guide the future direction of XR research in healthcare simulation. To organize these outcomes, questions were categorized into three types—descriptive, justification, and clarification—reflecting distinct stages of inquiry [18, 19]. Descriptive questions address the current landscape of XR use, capabilities, limitations, and applications within healthcare education. Justification questions seek evidence supporting XR’s educational value, such as its effectiveness in enhancing competency-based training or clinical skills acquisition. Clarification questions explore more nuanced issues, including learner characteristics, integration strategies, and contextual factors influencing successful adoption. Together, these categories provide a structured framework for advancing evidence-based implementation of XR in healthcare simulation, guiding researchers from foundational description to validation and refinement of practice.

## Theme 1: uses and adoption of extended reality (Table 3)

**Table 3:** Uses and adoption of XR in healthcare education

Category	Research Question	Type of Research
	**Technical Specifications**	
**1.1Technology and Definitions**	1.1.1-What are the key requirements and specifications for XR in healthcare education, including technical capabilities and alignment with desired learning outcomes?	Description
	1.1.2—How does adherence to these specifications affect the effectiveness, scalability, and overall user experience of XR in healthcare education?	Justification
	**Learner competencies and profiles**	
	1.1.3—What competencies would particularly benefit from XR (consensus-oriented research, based on clinical and educational needs, constrained by technological allowances)?	Description
	1.1.4—What is the profile of a learner who can most benefit from the use of XR?	Clarification
	1.1.5—What learning modalities are better suited to specific learner profiles?	Clarification
	1.1.6—Who is the “End User” and who are the different stakeholders in the integration of XR into healthcare education?	Description
	1.1.7—How can collaboration between educational researchers, clinicians, and XR developers establish standards for achieving and measuring competency?	Clarification
**1.2 Economic Considerations**	1.2.1—Which pricing models most effectively support the sustainable and equitable integration of XR into educational and clinical environments?	Clarification
	1.2.2—What is the resource effectiveness of XR in healthcare training, considering human, financial, physical, and environmental factors?	Justification
	**Faculty Development**	
**1.3 Implementation and Scalability**	1.3.1—What key competencies and skills should educators possess to effectively integrate XR into healthcare education?	Description
	1.3.2—How can faculty development programs be designed to address identified needs, bridging current competency gaps and equipping future educators with the required skills?	Clarification
	**Scalability and Sustainability**	
	1.3.3—How can education be scaled in a learner-directed/supported manner to accelerate mastery performance?	Justification
	**Impact/Effectiveness**	
**1.4 Educational Impact and Quality**	1.4.1—What are the unique educational value propositions of different XR systems (AR, VR, MR) in medical education and training?	Descriptive
	1.4.2—What are learner outcomes that can be measured using XR that are predictive of successful clinical performance?	Clarification
	1.4.3—What metrics should be included to assess the learning outcomes of XR in comparison to other learning methods ensuring a comprehensive evaluation of its effectiveness and added value?	Clarification
	**Enhance Clinical Training**	
**1.4 Educational Impact and Quality**	1.4.4—To what extent are skills learned using XR systems transferable to real-world clinical practice?	Justification
	1.4.5—How can the transferability of skills from XR-based training to clinical environments be effectively measured?	Clarification
	1.4.6—How do XR systems compare in fostering different skill sets (e.g., cognitive, technical, and non-technical skills)?	Descriptive
	1.4.7—How do learners perceive the impact of XR-enhanced simulations on their ability to retain and apply knowledge in emotionally charged clinical scenarios?	Clarification
	1.4.8—What are the effects of XR on learners’ cognitive flexibility and decision-making under stress?	Clarification
	1.4.9—What methodologies can XR training use to achieve higher levels of skill acquisition (e.g., problem-solving, critical thinking) beyond mere visibility and familiarity?	Description
	**Data-Driven Decision Making**	
	1.4.10—What framework can be established for data collection to evaluate the effectiveness of XR in achieving specific learning outcomes ensuring reliability, validity, and actionable insights??	Description
	1.4.11—How can big data extracted from XR experiences inform the refinement of frameworks and matrices used to evaluate its effectiveness in achieving learning outcomes?	Clarification
**1.5 Integration with Traditional and Novel Approaches**	1.5.1—Do XR systems serve best as augmentation tools within broader educational systems, or can they independently meet learning objectives?	Clarification
	1.5.2—What are the perceived benefits and challenges of aligning XR scenarios with established educational frameworks, such as experiential or problem-based learning?	Description
	1.5.3—Is XR a more educationally effective and efficient approach than existing educational/simulation approaches?	Justification
	1.5.4—What are the more effective/efficient hybrid (XR & traditional) approaches?	Clarification
	1.5.5—What can be learned from sectors like the military and video gaming to develop specific training protocols leveraging XR?	Clarification
**1.6 Customization & Adaptability vs. Standardization**	1.6.1—What components of a designed scenario need to be adjustable or customizable to meet various needs of a learner/program? (ex. regional variation, curricular, programmatic specificity, accreditation standards)	Descriptive
	1.6.2—What balance is needed between customization & standardization to achieve feasible local XR that does not compromise learning?	Clarification
	1.6.3—How can XR be adapted for various needs including continuing professional development (CPD) and patient/caregiver education	Clarification
**1.7 Emotional and Ethical Considerations**	1.7.1—What emotional states are most commonly reported during XR simulations, and how do they influence perceived learning outcomes?	Description
	1.7.2—What strategies do learners and educators believe are most effective in fostering psychological safety in XR-enhanced simulations?	Description
	1.7.3—How do healthcare learners and educators describe their emotional experiences when engaging with XR in simulation-based education?	Description
	1.7.4—How does XR facilitate the development of emotional regulation skills in healthcare learners?	Clarification
	1.7.5—What role do debriefing and reflection play in managing the emotional impact of XR simulations on learners and educators?	Clarification
	1.7.6—How do learners perceive the impact of XR-enhanced simulations on their ability to retain and apply knowledge in emotionally charged clinical scenarios?	Description
	1.7.7—What are the effects of XR on learners’ cognitive flexibility and decision-making under stress?	Clarification
	1.7.8—What are the unique risk and protective factors related to psychological safety engendered by the various XR modalities? What are threat mitigation approaches to support?	Clarification
	1.7.9—What factors in XR that can lead to better learning states XR (e.g. “flow”). What factors can be detrimental to learning or mental health (task irrelevant stress)?	Clarification
	1.7.10—How can XR environments be designed to prevent academic dishonesty while maintaining immersive and engaging experiences?	Clarification
	1.7.11—What are the best practices for managing and protecting personal and sensitive data collected through XR platforms?	Description
	1.7.12—How do different stakeholders (students, educators, administrators) perceive privacy risks associated with XR, and what measures do they prioritize for mitigating these risks?	Clarification
	1.7.13—How can interventions be designed to ensure equitable access to XR technologies and mitigate disparities in educational outcomes?	Clarification
	1.7.14—How can XR be leveraged to maximize positive educational outcomes while minimizing potential harms or negative impacts on learners?	Clarification

Deliberations around the use and adoption of XR in healthcare simulation emphasized that meaningful progress requires understanding how these technologies are defined, implemented, and evaluated in educational practice. Seven subthemes emerged; each associated with key research questions designed to guide future inquiry and promote evidence-based adoption.

### Technology and definitions

Participants highlighted the need for clear, shared definitions of XR modalities and their educational applications. Research questions in this area focus on identifying which competencies and learner profiles benefit most from XR, and what technological specifications are required to support effective and scalable learning. These questions are critical because the absence of standard terminology and performance criteria limits comparison, interoperability, and progress across the field.

### Economic considerations

Ensuring the sustainability and equity of XR adoption demands rigorous study of its economic dimensions. Research questions explore pricing models, cost-effectiveness, and resource utilization, including human, financial, and environmental factors. Understanding these issues is essential to support informed policy, funding, and institutional investment decisions, ensuring XR’s benefits are accessible across diverse educational settings.

### Implementation and scalability

Key questions examine how XR can be implemented and scaled sustainably, focusing on faculty development, infrastructure, and organizational readiness. These areas are crucial because even effective technologies fail without adequate educator training and institutional capacity. Research in this domain will help identify strategies for expanding XR integration from pilot projects to broad educational practice.

### Educational impact and quality

This subtheme centers on evaluating how XR influences learning outcomes, clinical performance, and learner engagement. Research questions address how XR supports skill transfer, cognitive flexibility, and data-driven decision-making in education. These studies are vital to demonstrate XR’s added value beyond novelty, clarifying where, when, and for whom it truly enhances learning.

### Integration with traditional and novel approaches

Research in this area explores how XR complements or transforms existing educational models, such as experiential, problem-based, or hybrid learning. Understanding optimal combinations of XR with traditional modalities will guide educators in designing curricula that balance innovation with proven pedagogical foundations.

### Customization & adaptability vs. standardization

Questions here address how XR can be adapted to local needs while maintaining consistency and quality. Exploring the balance between customization and standardization is important to ensure flexibility across programs without sacrificing comparability or accreditation standards.

### Emotional and ethical considerations

Finally, participants emphasized the emotional and ethical dimensions of XR use. Research questions focus on how immersive experiences affect psychological safety, emotional regulation, and learning outcomes, as well as how privacy, equity, and integrity can be safeguarded. The principle of beneficence, to do good, must be a cornerstone in the deployment of XR in education. This includes ensuring that these technologies do not harm learners or patients and that their application indeed enhances educational outcomes. These inquiries are essential to ensure that XR innovations remain learner-centered, ethically grounded, and socially responsible.

Together, these subthemes outline a forward-looking research agenda that connects technological development to educational value, implementation feasibility, and ethical responsibility, ensuring XR adoption advances healthcare simulation in both quality and integrity.

## Theme 2: barriers for Xr adoption and implementation in health professions education (Table 4)

**Table 4:** Barriers for XR adoption and implementation in healthcare education

Category	Research Question	Type of Research
**2.1 Technical and Infrastructure Challenges**	2.1.1—How can XR technology overcome scalability and dissemination processes in healthcare training to allow comparability studies in a comprehensive manner?	Clarification
	2.1.2—How can existing hardware be adapted effectively for new and evolving XR applications without compromising performance?	Description
	2.1.3—What specific IT infrastructure enhancements are necessary to support the robust demands of XR technology in healthcare education?	Clarification
	2.1.4—What are the infrastructure and logistic challenges that can limit uptake, application, and scalable? What strategies can be used to overcome these challenges?	Clarification
	2.1.5—What are the minimum and optimal hardware and software requirements for implementing XR in different healthcare training contexts?	Clarification
	2.1.6—How can XR technologies be integrated with existing learning management systems to effectively track and assess learner performance, and what are the key metrics that support meaningful feedback?	Justification
	2.1.7—What financial, human, and material resources are required for implementation and sustainability of XR education?	Clarification
**2.2 Resource and Space Constraints**	2.2.1—How can financial, human, and material resources be optimized for the sustainable integration of XR technologies in healthcare education?	Clarification
	2.2.2—Is the investment in XR technologies justified by the outcomes in healthcare training?	Justification
	2.2.3—What are the effects of mitigation strategies such as “teleporting” on learning?	Justification
	2.2.4—What are the ideal space and resources constraints for XR training in different contexts, populations, competencies, modalities, etc.?	Clarification
	**Staff Training**	
	2.2.4—What is the role of the healthcare simulation staff in supporting the implementation or adaptation of scenarios for individual programs?	Description
	2.2.5—Do staff and learners having an XR certification lead to more effective and efficient learning?	Justification
	**Faculty Training**	
	2.2.6—What are possible faculty development strategies and competencies related to XR?	Clarification
	2.2.7—How well do educator competencies translate from traditional educational settings or physical simulation to XR technologies?	Clarification
	2.2.8—What are the essential competencies required to be a competent XR educator?	Clarification
	**Collaborative Engagement**	
	2.2.9—Can we use XR technologies to open up a collaborative community mindset for scenario creation and sharing?	Clarification
	2.2.10—How can XR technology support collaborative engagement with software designers, technology companies, simulation companies, and content experts?	Clarification
**2.3 Usability Issues and Effectiveness**	**Understanding Technology**	
	2.3.1—Can XR authentically replicate real-world stimuli or conditions?	Justification
	2.3.2—What are the contributors to cyber-sickness? How can cyber-sickness in XR be minimized?	Justification
	2.3.3—How can students be motivated to use XR technologies for skill development and maintenance?	Clarification
	2.3.4—What are the most appropriate feedback and debriefing strategies for different XR experiences (e.g. group XR, independent learning in XR)?	Clarification
	2.3.5—How can we be confident in the validity of particular metrics used to measure particular competencies in XR systems?	Justification
	**User Accessibility and Content Limitations**	
	2.3.6—What are the best ways of interfacing with XR technologies when learning particular skills?	Description
	2.3.7—How are the barriers to uptake and use of VR related to context and the nature of technological advancement?	Clarification
	2.3.8—What types of XR modalities are efficient (or not) for different learners (e.g. neurodivergence) and contexts (e.g. low resource contexts)?	Clarification
**2.4 Psychological and Safety Concerns**	2.4.1—What is the relationship between XR technologies and perceptions of psychological safety in different contexts?	Clarification
	2.4.2 -What are the unique risk factors related to psychological safety engendered by the various XR modalities? What are threat mitigation approaches to support safety?	Clarification
	2.4.3—What strategies increase or decrease a sense of psychological safety?	Clarification
	2.4.4—How do learners feel a sense of pressure from observation, or freedom to immerse in the environment? How does this compare to other forms of simulation?	Clarification
	2.4.5—What data (e.g., usage, biometrics, location, performance) can XR data explicitly and implicitly collect, and what are the intended and unintended consequences?	Clarification
	2.4.6—How do prior experiences with XR technologies (e.g., gaming, social VR, or consumer AR) influence trainees' expectations, comfort, and psychological safety within XR-based clinical training environments?	Clarification
	2.4.7—In what ways can XR-based clinical training environments leverage positive aspects of gaming or entertainment XR design (e.g., feedback loops, user control, personalization) to enhance psychological safety and learner engagement?	Clarification
	2.4.8—How do expectations shaped by prior XR use influence trust in XR systems for clinical education (e.g., expectations about realism, accuracy, agency, or data privacy)?	Clarification

Discussions around barriers to XR adoption revealed that while enthusiasm for XR in healthcare education is growing, its widespread use remains constrained by several practical and human factors. Four major subthemes emerged, highlighting priority areas for research to guide sustainable and equitable implementation.

### Technical and Infrastructure challenges

Participants identified the technical and infrastructural requirements of XR as foundational barriers to its adoption at scale. Research questions in this domain focus on understanding the hardware, software, and IT infrastructure necessary to support high-performance, stable XR experiences across diverse institutions. These inquiries are essential because without clarity on minimum technical standards, equitable implementation across resource settings is not possible. Additional questions examine the costs associated with continuous hardware upgrades and the long-term sustainability of XR systems—issues that directly influence scalability, planning, and institutional commitment.

### Human resource and space constraints

Adopting XR in healthcare education also depends on adequate human and physical resources. Key questions address how best to prepare educators, simulation specialists, and technical staff to integrate XR into existing curricula, and what competencies are needed to sustain effective use. Faculty readiness remains one of the most significant determinants of success: without training, XR can remain underutilized or misapplied. Research also calls for exploration of collaborative partnerships among developers, educators, and content experts to optimize resource sharing, design, and deployment. These studies are important to ensure that XR adoption is not limited to well-resourced institutions but supported by scalable, collaborative models.

### Usability issues and effectiveness

Even when XR is available, usability challenges can impede its effective use. Research questions in this subtheme focus on optimizing user experience, minimizing cybersickness, improving accessibility, and validating metrics for performance assessment. Understanding how XR can authentically replicate real-world clinical settings, and how different learner populations engage with it, is essential for improving educational quality and inclusivity. These questions matter because poor usability or limited accessibility can erode learner confidence and undermine the perceived legitimacy of XR as an educational tool.

### Psychological and safety concerns

Finally, participants emphasized the importance of psychological safety in XR-based learning environments. Key research questions examine how immersive experiences influence learner anxiety, engagement, and perception of safety, as well as how prior experiences with gaming or virtual environments shape responses to XR in clinical training. Addressing these questions is critical to ensuring that XR promotes curiosity, reflection, and confidence rather than stress or disengagement. Understanding these psychological dimensions will guide best practices for scenario design, debriefing, and learner support.

Collectively, these subthemes and their associated research questions underscore that the barriers to XR adoption extend beyond technology, they include human, institutional, and psychological factors that must be addressed to ensure equitable, effective, and sustainable integration of XR in healthcare education.

## Theme 3: facilitators of the implementation and sustainability of Xr in healthcare education (Table 5)

**Table 5:** Facilitators of implementation and sustainability of XR in healthcare education

Category	Research Question	Type of Research
**3.1 Technical Support and Customization**	3.1.1—What strategies can be implemented to strengthen support mechanisms to tackle technical challenges in XR environments?	Clarification
	3.1.2—How can tools for tailoring XR content to specific educational needs be developed and utilized effectively?	Clarification
	3.1.3—How can game elements be utilized to enhance engagement and learning outcomes in XR training programs?	Clarification
**3.2 Resource Optimization and Skills Development**	**Resource Allocation**	
	3.2.1—What are effective strategies for allocating necessary resources to support XR implementations?	Clarification
	**Faculty Development**	
	3.2.2—What are effective training programs for educators to use and promote XR technologies efficiently?	Clarification
	3.2.3—What key competencies and skills should educators possess to effectively integrate XR into healthcare education?	Clarification
	**Staff Development**	
	3.2.4—What are the necessary skills for staff to manage and facilitate XR technologies	Description
	,3.2.5—How can the skills for staff to manage and facilitate XR be effectively developed?	Clarification
**3.3 Empirical Evaluation**	3.3.1—How can data collection and analysis be utilized to validate the effectiveness of XR compared to traditional methods?	Justification
	3.3.2—Can XR environments be used effectively for summative assessments, such as OSCEs?	Justification
	3.3.3—How predictive are XR-based assessments of real-world clinical performance?	Justification
	3.3.4—How do skills learned using XR technologies transfer to real-world clinical practice??	Justification
	3.3.5—How does XR enhance learners’ abilities to interact with patients in real-world clinical environments	Clarification
	3.3.6—How can data-driven metrics from XR experiences guide learner performance, ensuring reliable and actionable feedback?	Clarification
	3.3.7—How can data-driven metrics guide the design and implementation of curricula to maximize learning outcomes and integration into existing educational frameworks?	Clarification
	3.3.8—How can data-driven metrics guide iterative improvements and innovations in technology to better meet the needs of healthcare education?	Clarification
**3.4 Learning from Past Experiences –** **Failures and Successes**	3.4.1—How can historical insights from other technologies guide XR development and adoption strategies in healthcare education?	Clarification
	3.4.2—What are educational techniques from other industries that apply to HR healthcare application (arts, military, gaming, etc.)?	Clarification
	3.4.3—Do educational techniques from other industries translate to more effective and efficient HR training in healthcare?	Justification
	3.4.4—How can education techniques from other industries be modified and optimized for use in XR applications	Clarification
	3.4.5—How can optimal partnerships between academia and industry be established to enhance XR applications in healthcare education?	Clarification
**3.5 Cross-Cutting Themes and Future Focus Areas**	**Scalability and Sustainability**	
	3.5.1—How can XR technologies be scaled to support wider adoption without compromising quality or increasing costs?	Clarification
	**Enhancing User Experience and Safety**	
	3.5.2—How can solutions be investigated and implemented to improve psychological safety and user comfort during XR sessions?	Clarification
	3.5.3—How does XR facilitate psychologically safe learning environments compared to traditional simulation methods?	Justification
	3.5.4—Can XR reduce learner vulnerability in small-group, high-stakes simulations, particularly for senior clinicians revisiting rarely used skills?	Justification
	**Integrating XR with Traditional Learning**	
	3.5.5—How can hybrid models that incorporate XR alongside conventional training methods be developed to provide a balanced educational experience?	Clarification
	3.5.6—Where does XR fit in in a sequential learning pathway, ranging from simulated to real patient encounters	Clarification
	3.5.7—How does XR support the scaffolding of competencies in healthcare education?	Clarification
	3.5.8—Which educational theories best support the implementation of XR in healthcare training, and how do these theories align with observed outcomes?	Clarification
	3.5.9—What are the educational benefits of assigning students virtual patients for longitudinal interaction and decision-making in XR environments?	Clarification
	3.5.10—How does the "longitudinal virtual patient" approach compare to traditional patient exposure in fostering clinical decision-making skills?	Justification
	**Customization and Accessibility**	
	3.5.11—How can XR tools be made more customizable and accessible to a broader range of educational institutions and settings?	Description
	3.5.12—How does the ability to customize XR environments to reflect diverse profiles influence learner engagement and inclusivity?	Clarification
	3.5.13—Does customization of avatars and environments impact the sense of community and collaboration among learners?	Clarification
	3.5.14—What are the key requirements and specifications for XR in healthcare education that ensure adaptability to learner needs while optimizing the allocation and utilization of available resources?	Description
	3.5.15—How does XR support self-regulated learning through customizable content and adaptive systems?	Clarification
	3.5.16—Is there a measurable difference in learner outcomes when using adaptive XR systems as compared to educator-directed methods?	Justification
	3.5.16—How do cultural differences affect the adoption and effectiveness of XR in healthcare education?	Clarification
	3.5.17—How does the representation of avatars (e.g., realistic, abstract, or customizable) in XR environments influence learner engagement and educational outcomes?	Clarification
	3.5.18—How does an avatar’s appearance impact learners' psychological connection to virtual patients or peers?	Clarification

Deliberations on facilitators of XR focused on identifying enabling conditions that promote effective integration and sustainability. Five subthemes emerged, outlining key strategies and research priorities that can accelerate the responsible and evidence-based use of XR in health professions education.

### Technical support and customization

Participants emphasized that robust technical support and adaptable XR platforms are critical to overcoming implementation barriers. Research questions focus on how best to structure support systems to ensure reliable performance and how customization tools can align XR experiences with specific curricular objectives. Gamification was also identified as an area for exploration to increase engagement and motivation. These lines of inquiry are important because they address the practical mechanisms by which XR can move from isolated innovation to dependable educational infrastructure.

### Resource optimization and skill development

Effective use of XR depends on optimizing institutional resources and investing in faculty and staff expertise. Key research questions examine how resources can be allocated most efficiently and how targeted faculty development programs can build the competencies needed for XR-enhanced teaching and learning. These questions are significant because sustainable adoption requires not only technology but also people who can use it confidently and creatively.

### Empirical evaluation

Empirical evaluation was recognized as foundational to prove XR’s educational value. Research questions explore how XR can be integrated into formative and summative assessments, including Objective Structured Clinical Examinations (OSCEs), and how its outcomes predict real-world clinical performance. Additional questions address how XR-generated data can inform feedback, curriculum design, and iterative improvement. This work is essential to demonstrate XR’s measurable impact, move beyond anecdotal claims, and support data-driven decision-making in education.

### Learning from past experiences – failures and successes

Participants highlighted the importance of reflecting on previous technological adoptions to guide future XR integration. Research questions address what can be learned from both the successes and missteps of earlier innovations in healthcare, gaming, and military training. Understanding these lessons is vital to avoid repeating past challenges, accelerate adoption, and adapt proven strategies to the unique demands of healthcare education.

### Cross-cutting themes and future focus areas

Finally, several cross-cutting facilitators emerged that span multiple domains. Research questions address how XR can enhance user experience and psychological safety, support inclusive participation, and integrate effectively with traditional educational approaches. Hybrid and longitudinal learning models, combining XR with conventional methods, were identified as promising directions for future study. These areas are critical because they emphasize that effective facilitation is not merely technical, it involves aligning innovation with pedagogy, accessibility, and learner well-being.

Together, these subthemes and research questions provide a forward-looking framework for strengthening the conditions under which XR can flourish in healthcare education. By focusing on facilitation, institutions can move from experimentation to sustained integration, ensuring XR technologies fulfill their promise to advance learning, equity, and clinical preparedness.

#### Implications for research and practice

The research priorities identified through this Utstein style meeting provide a practical roadmap for advancing the integration of XR in healthcare education. Future studies should apply implementation-science frameworks to evaluate how XR can be effectively adopted, scaled, and sustained across diverse contexts. Rigorous empirical work is needed to establish educational impact, cost-effectiveness, and faculty readiness, ensuring that XR enhances existing simulation approaches. For practice, the agenda underscores the importance of institutional investment in infrastructure, training, and policy alignment to enable equitable, evidence-informed use of XR technologies in health professions education. The research agenda generated through this process also provides a structured foundation for advancing the science of XR in healthcare simulation, offering targeted questions to explore both its immediate applications and long-term implications for professional education.

## Conclusions

This Utstein style meeting represents a structured, consensus-driven effort to establish a research agenda for the use of extended reality (XR) in healthcare simulation. Through a collaborative and multidisciplinary process, participants identified key priorities that define how XR can best enhance health professions education and improve learner outcomes. The meeting’s outcome demonstrative XR’s potential to provide immersive, interactive, and scalable learning environments that can complement and extend traditional simulation methods.

Discussions highlighted the need for focused research across several domains, including technological development, economic feasibility, implementation strategies, and educational impact, to guide evidence-based adoption. At the same time, participants identified significant barriers such as technical limitations, infrastructure requirements, and human resource constraints that must be addressed to ensure equitable and sustainable integration.

Importantly, the research agenda aligns with established implementation-science frameworks, particularly the NASSS model. The three core themes, uses and adoption, barriers, and facilitators, reflect a continuum of inquiry from defining XR’s educational value, to understanding implementation challenges, to identifying enablers of sustainability. This alignment ensures that future XR research moves beyond describing technological innovation to examining how it is adopted, scaled, and sustained within complex educational systems. Integrating this implementation-science perspective provides a structured pathway for linking theoretical understanding with practical strategies that can inform institutional policy, faculty development, and long-term impact (Table 6).

**Table 6:** XR research agenda aligned with implementation science framework

Implementation Science Theme	Subthemes	Core Research Focus	Why It Matters
Adoption & Use	Technology & Definitions	Define XR modalities, competencies, and learner profiles to ensure consistency and alignment with educational goals	Establishing shared definitions and competency targets ensures interoperability, standardization, and meaningful comparison of results across studies, enabling consistent implementation and evaluation of XR-based education
	Economic Considerations	Identify sustainable pricing models, cost-effectiveness measures, and equitable resource distribution strategies	Economic evidence supports decision-making for policy, funding, and institutional investment, addressing feasibility and sustainability barriers across diverse educational settings
	Implementation & Scalability	Explore how XR can be implemented and scaled across curricula through faculty development, infrastructure support, and institutional readiness	Implementation-focused research ensures that XR adoption moves beyond pilot programs to sustainable, system-level integration supported by skilled educators and robust infrastructure
Barriers to Adoption	Technical & Infrastructure Challenges	Identify system requirements, IT and hardware standards, and strategies to overcome scalability and resource limitations	Clarifying minimal and optimal infrastructure needs and cost implications enables equitable implementation and broader access to XR technologies globally
	Human Resource & Space Constraints	Define essential educator competencies and staff training models for effective XR use and integration	Human resource preparedness is critical to ensuring XR’s educational potential is realized; faculty training is a central determinant of adoption and sustainability
	Usability & Safety	Address user experience, accessibility, and psychological safety concerns in XR environments	Research that reduces usability and safety barriers improves learner engagement, inclusivity, and trust in XR technologies, ensuring educational environments are both effective and safe
Facilitators for Integration & Sustainability	Technical Support & Customization	Develop models for continuous technical support and tools for customizing XR experiences to curricular needs	Reliable support systems and customization capabilities enhance educator confidence, user experience, and relevance of XR to diverse learning contexts
	Empirical Evaluation	Establish data-driven methods to evaluate learning outcomes, skill transfer, and real-world clinical performance using XR	Empirical validation strengthens the evidence base for XR, guiding evidence-informed adoption, institutional investment, and accreditation alignment
	Learning from Past Experiences	Translate lessons from prior technology adoptions and other industries (e.g., gaming, aviation) into XR implementation	Applying lessons from other fields accelerates adoption, minimizes risk, and leverages proven strategies for integrating innovation into education
	Cross-Cutting Themes (User Experience, Equity, and Ethics)	Explore how inclusivity, cultural context, emotional safety, and ethical practices influence adoption and learner engagement	Embedding ethics, equity, and psychological safety principles within implementation strategies ensures XR integration remains learner-centered, sustainable, and socially responsible

Ultimately, the outcomes of this Utstein style meeting highlight XR’s capacity to transform healthcare education through innovation grounded in evidence and collaboration. By pursuing the research priorities identified here, the simulation community can help realize XR’s full potential to enhance training, improve competency, and expand access to high-quality healthcare education worldwide.

## Supplementary Information

Supplementary Material 1.

Supplementary Material 2.

Supplementary Material 3.

Supplementary Material 4.

## Data Availability

The datasets used and/or analysed during the current study are available from the corresponding author on reasonable request.
