# Supplementary material for: Setting a research agenda for the use of extended reality in healthcare simulation: an Utstein style meeting
Source: Adv Simul (Lond). 2026 Mar 3;11:16. doi: 10.1186/s41077-026-00409-y (PMC12954913; doi:10.1186/s41077-026-00409-y)
Supplement: Supplementary file 1 — Supplementary Material 1. [file 41077_2026_409_MOESM1_ESM.docx]

**Appendix 1 - Reviews on VR, AR, MR, and XR in Healthcare and Medical Education**

Alnagrat, A., Che Ismail, R., Syed Idrus, S. Z., & Abdulhafith Alfaqi, R. M. (2022). A Review of Extended Reality (XR) Technologies in the Future of Human Education: Current Trend and Future Opportunity. Journal of Human Centered Technology, 1(2), 81–96. https://doi.org/10.11113/humentech.v1n2.27

Barteit S., Lanfermann L., Bärnighausen T., Neuhann F., Beiersmann C. Augmented, Mixed, and Virtual Reality-Based Head-Mounted Devices for Medical Education: Systematic Review. JMIR Serious Games. 2021 Jul 8;9(3):e29080. doi: 10.2196/29080. PMID: 34255668; PMCID: PMC8299342.

Barsom EZ., Graafland M., Schijven MP. Systematic review on the effectiveness of augmented reality applications in medical training. Surg Endosc. 2016 Oct;30(10):4174-83. doi: 10.1007/s00464-016-4800-6. Epub 2016 Feb 23. PMID: 26905573; PMCID: PMC5009168.

Co M., Chiu S., Billy Cheung HH. Extended reality in surgical education: A systematic review. Surgery. 2023 Nov;174(5):1175-1183. doi: 10.1016/j.surg.2023.07.015. Epub 2023 Aug 26. PMID: 37640664.

Curran VR, Xu X, Aydin MY, Meruvia-Pastor O. Use of Extended Reality in Medical Education: An Integrative Review. Med Sci Educ. 2022 Dec 19;33(1):275-286. doi: 10.1007/s40670-022-01698-4. PMID: 36569366; PMCID: PMC9761044.

Gasteiger N., van der Veer S., Wilson P., Dowding D. How, for Whom, and in Which Contexts or Conditions Augmented and Virtual Reality Training Works in Upskilling Health Care Workers: Realist Synthesis. JMIR Serious Games 2022;10(1):e31644. URL: https://games.jmir.org/2022/1/e31644. DOI: 10.2196/31644.

Gerup J., Soerensen CB., Dieckmann P. Augmented reality and mixed reality for healthcare education beyond surgery: an integrative review. Int J Med Educ. 2020 Jan 18;11:1-18. doi: 10.5116/ijme.5e01.eb1a. PMID: 31955150; PMCID: PMC7246121.

Gómez Bergin, A.D., Craven, M.P. Virtual, augmented, mixed, and extended reality interventions in healthcare: a systematic review of health economic evaluations and cost-effectiveness. BMC Digit Health 2023. 1, 53. https://doi.org/10.1186/s44247-023-00054-9

Herur-Raman, A., Almeida, N., Greenleaf, W., Williams, D., Karshenas, A., Sherman, J. Next-Generation Simulation—Integrating Extended Reality Technology Into Medical Education. Frontiers in Virtual Reality. 2021. 2. 10.3389/frvir.2021.693399.

Iop A., El-Hajj VG., Gharios M., de Giorgio A., Monetti FM., Edström E., Elmi-Terander A., Romero M. Extended Reality in Neurosurgical Education: A Systematic Review. Sensors (Basel). 2022 Aug 14;22(16):6067. doi: 10.3390/s22166067. PMID: 36015828; PMCID: PMC9414210.

Jensen, L., Konradsen, F. A review of the use of virtual reality head-mounted displays in education and training. Education and Information Technologies. 2018. 23. 1-15. 10.1007/s10639-017-9676-0.

Kyaw BM., Saxena N., Posadzki P., Vseteckova J., Nikolaou CK., George PP., Divakar U., Masiello I., Kononowicz AA., Zary N., Tudor Car L. Virtual Reality for Health Professions Education: Systematic Review and Meta-Analysis by the Digital Health Education Collaboration. J Med Internet Res. 2019 Jan 22;21(1):e12959. doi: 10.2196/12959. PMID: 30668519; PMCID: PMC6362387.

Logeswaran A., Munsch C., Chong YJ., Ralph N., McCrossnan J. The role of extended reality technology in healthcare education: Towards a learner-centred approach. Future Healthc J. 2021 Mar;8(1):e79-e84. doi: 10.7861/fhj.2020-0112. PMID: 33791482; PMCID: PMC8004346.

Ong CW., Tan MCJ., Lam M., Koh VTC. Applications of Extended Reality in Ophthalmology: Systematic Review. J Med Internet Res. 2021 Aug 19;23(8):e24152. doi: 10.2196/24152. PMID: 34420929; PMCID: PMC8414293.

Sadek O., Baldwin F., Gray R., Khayyat N., Fotis T. Impact of Virtual and Augmented Reality on Quality of Medical Education During the COVID-19 Pandemic: A Systematic Review. J Grad Med Educ. 2023 Jun;15(3):328-338. doi: 10.4300/JGME-D-22-00594.1. Epub 2023 Jun 14. PMID: 37363680; PMCID: PMC10286921.

Sheik-Ali S., Edgcombe H., Paton C. Next-generation Virtual and Augmented Reality in Surgical Education: A Narrative Review. Surg Technol Int. 2019 Nov 10;35:27-35. PMID: 31498872.

Stretton T., Cochrane T., & Narayan V. Exploring mobile mixed reality in healthcare higher education: A systematic review. Research in Learning Technology, 2018. 26. https://doi.org/10.25304/rlt.v26.2131

Taylor L., Dyer T., Al-Azzawi M., Smith C., Nzeako O., Shah Z. Extended reality anatomy undergraduate teaching: A literature review on an alternative method of learning. Ann Anat. 2022 Jan;239:151817. doi: 10.1016/j.aanat.2021.151817. Epub 2021 Aug 12. PMID: 34391910.

Towers A., Field J., Stokes C., Maddock S., Martin N. A scoping review of the use and application of virtual reality in pre-clinical dental education. Br Dent J. 2019 Mar;226(5):358-366. doi: 10.1038/s41415-019-0041-0. PMID: 30850794.

Woodall WJ., Chang EH., Toy S., Lee DR., Sherman JH., Liu M., Chen P., Youner E., Cooke J., Lancaster A., Gerberi D., Herur-Raman A. Does Extended Reality Simulation Improve Surgical/Procedural Learning and Patient Outcomes When Compared With Standard Training Methods?: A Systematic Review. Simul Healthc. 2024 Jan 1;19(1S):S98-S111. doi: 10.1097/SIH.0000000000000767. PMID: 38240622.

Xu, X., Mangina, E., Campbell, A. HMD-Based Virtual and Augmented Reality in Medical Education: A Systematic Review. 2021. 2. 82. 10.3389/frvir.2021.692103.
