# Supplementary material for: Setting a research agenda for the use of extended reality in healthcare simulation: an Utstein style meeting
Source: Adv Simul (Lond). 2026 Mar 3;11:16. doi: 10.1186/s41077-026-00409-y (PMC12954913; doi:10.1186/s41077-026-00409-y)
Supplement: Supplementary file 2 — Supplementary Material 2. [file 41077_2026_409_MOESM2_ESM.docx]

**Utstein Summit Goals – Areas of Focus and Priority**

**For each item listed, please** rate the importance/priority of each item on a scale (e.g., 1 to 5, where 1 is least important and 5 is most important)

| **Goal 1: Identify the key elements that influence the adoption new XR programs – what gap/opportunity is XR being used to address/realize?** | |
| --- | --- |
| **Gap/Opportunity** | **Priority** |
| **Enhance Clinical Training:** XR provides an immersive platform for clinical training where medical students and professionals can practice complex procedures in a controlled and risk-free virtual environment. This hands-on experience is invaluable for building competence and confidence before performing procedures on actual patients. |  |
| **Improved Access to Training:** XR can make high-quality training accessible to a wider audience, including healthcare learners and providers in remote or underserved areas. This technology can eliminates geographical barriers, allowing for consistent training standards across different locations and institutions. |  |
| **Cost-Effective Simulation:** Traditional training simulations can be prohibitively expensive due to the need for specialized equipment and facilities. XR simulations reduce these costs by virtualizing expensive resources and equipment, making training more affordable and scalable. |  |
| **Standardization of Training:** XR programs can be designed to follow specific training protocols, ensuring that all learners receive the same level of training and experience. This standardization is crucial for maintaining high educational standards and ensuring that all healthcare professionals are adequately prepared. |  |
| **Realistic Scenarios for Rare/Special Situations:** XR allows trainers to create highly realistic and diverse scenarios that healthcare providers might encounter, such as mass casualty incidents or rare medical conditions. This preparation is critical for improving responses in actual emergencies. |  |
| **Interactive and Engaging Learning:** By leveraging gamification and interactive elements, XR makes learning more engaging and enjoyable for users. This interactivity can lead to higher engagement rates, improved motivation, and better learning outcomes. |  |
| **Detailed Performance Feedback:** XR technologies can track a user’s actions in detail, providing precise feedback that can help learners identify and correct mistakes. This feedback is vital for refining skills and improving clinical performance. |  |
| **Collaborative Learning and Telementoring:** XR enables real-time collaboration and telementoring, where experienced practitioners can guide less experienced colleagues through complex procedures from remote locations, enhancing learning opportunities and patient care. |  |
| **Patient Education and Engagement:** XR can transform patient education by providing interactive and understandable visualizations of medical conditions and treatments. This enhanced understanding can lead to better patient engagement and compliance with treatment protocols. |  |
| **Research and Development:** XR can be used to study complex biological processes in a virtual environment, facilitate the understanding of drug interactions within the body, or simulate the progression of diseases. It offers new possibilities for conducting controlled experiments and hypothesis testing without ethical concerns or logistical constraints. |  |
| **Comments/Suggestions for Other Topic Areas not Listed Above:** |  |

| **Goal 2a: Identify the Barriers of the Use and Implementation of These Programs** | |
| --- | --- |
| **Barriers Identified** | **Priority** |
| **Technological Limitations** |  |
| **Infrastructure:** Lack of necessary hardware and software infrastructure can hinder the adoption of XR. This includes insufficient computing power, lack of VR-ready devices, and inadequate network capabilities. |  |
| **Usability Issues:** Complexities in using XR systems, such as user-unfriendly interfaces or cumbersome equipment, can deter faculty and students from fully engaging with the technology. |  |
| **Cybersickness and Physical Discomfort:** Symptoms like nausea, eye strain, and disorientation while using XR can limit prolonged use and negatively affect learning experiences. |  |
| **Financial Constraints** |  |
| **High Initial costs** |  |
| **Infrastructure and Hardware:** Upgrading existing IT infrastructure to support the data and graphical requirements of XR technologies often requires significant investment. XR hardware, including VR headsets, AR glasses, and associated peripherals, can be expensive, especially when purchasing enough units to support large classes. |  |
| **Software and Updates**: Licensing fees for high-quality XR software or custom development costs for specific educational applications can be substantial. Continuous software updates are necessary to keep XR applications functional and secure, which can incur ongoing costs. |  |
| **Scaling Challenges** |  |
| **Expansion Costs:** Scaling XR implementations from pilot projects to full integration across multiple courses or departments can be financially demanding. |  |
| **Sustainability:** Ensuring the long-term sustainability of XR initiatives, including funding for ongoing support staff and technology refresh cycles, can strain budgets. |  |
| **Cultural and Organizational Resistance** |  |
| **Resistance to Change:** Faculty and administrative resistance to integrating new technologies into traditional curricula can be a significant barrier. |  |
| **Lack of Awareness and Understanding:** Insufficient knowledge about the benefits and potential applications of XR in education can result in underutilization. |  |
| **Regulatory and Ethical Issues** |  |
| **Privacy Concerns:** Handling sensitive data within XR environments poses privacy risks that must be addressed through strict data management protocols. |  |
| **Ethical Use:** Ethical dilemmas, such as the potential for bias in virtual scenarios or the psychological impact of immersive simulations, need careful consideration. |  |
| **Comments/Suggestions for Other Topic Areas not Listed Above:** |  |

| **Goal 2b: Identify the Facilitators of the Use and Implementation of These Programs** | |
| --- | --- |
| **Facilitators Identified** | **Priority** |
| **Strategic Partnerships** |  |
| **Collaborations with Technology Providers:** Partnering with XR hardware and software vendors can facilitate access to cutting-edge technology and technical support. |  |
| **Academic and Industry Partnerships:** Collaborations can provide mutual benefits, including shared resources, expertise, and funding opportunities. |  |
| **Policy and Framework Development** |  |
| **Institutional Policies:** Developing clear policies that support the adoption and ethical use of XR technologies can provide a structured framework for integration. |  |
| **Curricular Integration Guidelines:** Guidelines that help faculty incorporate XR into the curriculum systematically can facilitate smoother adoption. |  |
| **Evidence-based Practice** |  |
| **Research and Case Studies:** Demonstrating the effectiveness of XR through research and successful case studies can help overcome skepticism and build a case for wider adoption. |  |
| **Outcome Measures:** Robust metrics to evaluate the impact of XR on learning outcomes can justify investment and encourage broader use. |  |
| **Comments/Suggestions for Other Topic Areas not Listed Above:** |  |

| **Goal 3: A Faculty Development Program in Healthcare XR** | |
| --- | --- |
| **Area of Need** | **Priority** |
| **Technological Proficiency**: |  |
| **Training**: Faculty members need comprehensive training on the technical aspects of XR technologies. This includes understanding the hardware and software, troubleshooting common issues, and staying updated on the latest developments. |  |
| **Skill Development**: Developing skills for effectively integrating XR tools into the educational process. This includes scenario design, instructional design tailored to XR, and adapting teaching styles to immersive learning environments. |  |
| **Pedagogical Integration**: |  |
| **Curriculum Development**: Designing curricula that incorporate XR in a way that enhances learning outcomes. Faculty should understand how to use XR to complement traditional teaching methods rather than replace them. |  |
| **Instructional Design**: Training on how to create engaging and educationally effective XR experiences. Developing competencies and teaching methodologies that leverage XR technologies effectively (scenario-based learning, gamification, interactive design principles). |  |
| **Assessment and Evaluation**: |  |
| **Outcome Measures**: Educating faculty on how to measure and evaluate the impact of XR on learning outcomes. This includes understanding which metrics are most relevant for XR-based education and how to collect and analyze this data. |  |
| **Continuous Improvement**: Training faculty to use feedback and data from XR sessions to refine & improve the educational content & delivery |  |
| **Ethical and Practical Considerations**: |  |
| **Accessibility and Inclusivity**: Ensuring faculty are aware of and know how to address potential accessibility issues with XR, making the technology inclusive for all students. |  |
| **Ethical Use of Technology**: Understanding the ethical implications of using immersive technology in education, including privacy concerns and the psychological impact on students. |  |
| **Comments/Suggestions for Other Topic Areas not Listed Above:** |  |

| **Goal 4: Translate the Actual Knowledge Gaps into a Research Agenda for the Next 3 Years** | |
| --- | --- |
| **Area of Focus** | **Priority** |
| **Effectiveness and Impact:** |  |
| Develop and validate measures to assess the effectiveness and impact of XR applications. |  |
| Comparative studies to establish the efficacy of different XR modalities. |  |
| **Outcome Measures:** |  |
| Validation of study outcomes and effectiveness of XR in changing clinical practice and behavior. |  |
| **Technological Challenges** |  |
| Continued development and refinement of XR technologies to address identified user challenges and limitations. |  |
| **Translational Research Outcomes:** |  |
| Create actionable frameworks & guidelines for the sustainable implementation of XR in healthcare settings, especially in underserved regions. |  |
| **Methodological Rigor and Validation** |  |
| **Extent of valid use of extended reality applications**. Determining the extent of valid use involves identifying the specific educational settings and scenarios where XR provides significant learning benefits, which is currently underexplored and lacks comprehensive guidelines. |  |
| **Completion of full validation processes for technologies.** There is a gap in fully validating XR technologies through all stages of development and deployment, ensuring that they meet educational objectives and maintain effectiveness over time. |  |
| **Standardization and comparability of study designs.** The absence of standardized study designs in XR research leads to challenges in comparing results across different studies, hindering the synthesis of evidence and the development of generalizable conclusions. |  |
| **Need for higher-quality studies and robust evidence.** There is a critical need for higher-quality research studies that employ rigorous methodologies and robust evidence collection to substantiate the claims made about the educational impacts of XR technologies. |  |
| **Lack of validity in Study conclusions.** Many studies on XR applications suffer from validity issues in their conclusions, often due to methodological weaknesses, inadequate controls, or biases in data interpretation, which compromises their reliability and applicability. |  |
| **Comments/Suggestions for Other Topic Areas not Listed Above:** |  |
