# Supplementary material for: Setting a research agenda for the use of extended reality in healthcare simulation: an Utstein style meeting
Source: Adv Simul (Lond). 2026 Mar 3;11:16. doi: 10.1186/s41077-026-00409-y (PMC12954913; doi:10.1186/s41077-026-00409-y)
Supplement: Supplementary file 3 — Supplementary Material 3. [file 41077_2026_409_MOESM3_ESM.docx]

Overall Status

Number of answers

New 0


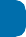


0%

0%

4%

96%

0%

Distributed 0

Partially Complete 1

Complete 22

Rejected 0

0% 25% 50% 75% 100%

Gap/Opportunity

**Enhance Clinical Training:** XR provides an immersive platform for clinical training where medical students and professionals can practice complex procedures in a controlled and risk-free virtual environment. This hands-on experience is invaluable for building competence and confidence before performing procedures on actual patients. **86%**

| 1 | 0% |  |  |  |  |  | Number of answers  0 |
| --- | --- | --- | --- | --- | --- | --- | --- |
| 2 | 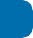 | 5% |  |  |  |  | 1 |
| 3 | 9% |  |  |  |  |  | 2 |
| 4 |  |  |  | 50% |  |  | 11 |
| 5 |  |  | 36% |  |  |  | 8 |
|  | 0% |  | 25% | 50% | 75% | 100% |  |

**Improved Access to Training:** XR can make high-quality training accessible to a wider audience, including healthcare learners and providers in remote or underserved areas. This technology can eliminate geographical barriers, allowing for consistent training standards across different locations and institutions. **81%**

| 1 | 0% |  |  |  |  | Number of answers  0 |
| --- | --- | --- | --- | --- | --- | --- |
| 2 | 9% |  |  |  |  | 2 |
| 3 | 9% |  |  |  |  | 2 |
| 4 |  | 36% |  |  |  | 8 |
| 5 |  |  | 45% |  |  | 10 |

0% 25% 50% 75% 100%

Cost-Effective Simulation: Traditional training simulations can be prohibitively expensive due to the need for specialized equipment and facilities. XR simulations reduce these costs by virtualizing expensive resources and equipment, making training more affordable and scalable. **59%**

| 1 | 0% |  |  |  |  | Number of answers  0 |
| --- | --- | --- | --- | --- | --- | --- |
| 2 |  | 18% |  |  |  | 4 |
| 3 |  | 23% |  |  |  | 5 |
| 4 |  |  | 41% |  |  | 9 |
| 5 |  | 18% |  |  |  | 4 |

0% 25% 50% 75% 100%

**Standardization of Training:** XR programs can be designed to follow specific training protocols, ensuring that all learners receive the same level of training and experience. This standardization is crucial for maintaining high educational standards and ensuring that all healthcare professionals are adequately prepared. **77%**

| 1 | 0% |  |  |  |  | Number of answers  0 |
| --- | --- | --- | --- | --- | --- | --- |
| 2 | 9% |  |  |  |  | 2 |
| 3 | 14% |  |  |  |  | 3 |
| 4 |  |  | 45% |  |  | 10 |
| 5 |  | 32% |  |  |  | 7 |

0% 25% 50% 75% 100%

**Realistic Scenarios for Rare/Special Situations:** XR allows trainers to create highly realistic and diverse scenarios that healthcare providers might encounter, such as mass casualty incidents or rare medical conditions. This preparation is critical for improving responses in actual emergencies. **73%**

Number of answers

1 0

0%

9%

18%

32%

41%

2 2

3 4

4 7

5 9

0% 25% 50% 75% 100%

**Interactive and Engaging Learning:** By leveraging gamification and interactive elements, XR makes learning more engaging and enjoyable for users. This interactivity can lead to higher engagement rates, improved motivation, and better learning outcomes. **64%**

| 1 | 0% |  |  |  |  |  | Number of answers  0 |
| --- | --- | --- | --- | --- | --- | --- | --- |
| 2 | 9% |  |  |  |  |  | 2 |
| 3 |  | 27% |  |  |  |  | 6 |
| 4 |  |  | 41% |  |  |  | 9 |
| 5 |  | 23% |  |  |  |  | 5 |
|  | 0% | 25% |  | 50% | 75% | 100% |  |

**Detailed Performance Feedback:** XR technologies can track a user’s actions in detail, providing precise feedback that can help learners identify and correct mistakes. This feedback is vital for refining skills and improving clinical performance. **82%**

Number of answers

1 0


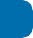


0%

5%

14%

50%

32%

2 1

3 3

4 11

5 7

0% 25% 50% 75% 100%

**Collaborative Learning and Telementoring:** XR enables real-time collaboration and telementoring, where experienced practitioners can guide less experienced colleagues through complex procedures from remote locations, enhancing learning opportunities and patient care. **68%**

| 1 | 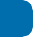 | 5% |  |  |  |  | Number of answers  1 |
| --- | --- | --- | --- | --- | --- | --- | --- |
| 2 | 9% |  |  |  |  |  | 2 |
| 3 |  | 18% |  |  |  |  | 4 |
| 4 |  |  | 36% |  |  |  | 8 |
| 5 |  |  | 32% |  |  |  | 7 |
|  | 0% |  | 25% | 50% | 75% | 100% |  |

**Patient Education and Engagement:** XR can transform patient education by providing interactive and understandable visualizations of medical conditions and treatments. This enhanced understanding can lead to better patient engagement and compliance with treatment protocols. **59%**

| 1 | 0% |  |  |  |  | Number of answers  0 |
| --- | --- | --- | --- | --- | --- | --- |
| 2 |  | 18% |  |  |  | 4 |
| 3 |  | 23% |  |  |  | 5 |
| 4 |  |  | 36% |  |  | 8 |
| 5 |  | 23% |  |  |  | 5 |

0% 25% 50% 75% 100%

**Research and Development:** XR can be used to study complex biological processes in a virtual environment, facilitate the understanding of drug interactions within the body, or simulate the progression of diseases. It offers new possibilities for conducting controlled experiments and hypothesis testing without ethical concerns or logistical constraints**. 50%**

| 1 | 14% |  |  |  |  | Number of answers  3 |
| --- | --- | --- | --- | --- | --- | --- |
| 2 | 9% |  |  |  |  | 2 |
| 3 |  | 27% |  |  |  | 6 |
| 4 |  | 27% |  |  |  | 6 |
| 5 |  | 23% |  |  |  | 5 |
|  | 0% | 25% | 50% | 75% | 100% |  |

| **Comments/Suggestions for Other Topic Areas not Listed Above:** |
| --- |
| XR enables the visualization of both anatomical structures and physiological processes, which can be seamlessly integrated into simulation environments. This promotes a comprehensive understanding at both macro and micro levels. |
| While I feel that many of these are important goals for XR, the technology is not yet at a point where it is either cost effective or advanced enough to provide the realism required to meet these goals.  XR simulation could also be used for mental health purposes - such as helping patients in rehabilitation programs as well as controlled and supported exposure to stress-inducing or potentially traumatic situations. |
| There is a great potential for XR in clinical training, however currently the technology is not quite there yet for psychomotor skills. There are opportunities for critical thinking and decision making, but it is a critical time to think about the process for increased clinical training when the technology surrounding hand haptics and psychomotor skills are improved. Cost may be an issue up front, but once an adopted program is in place, the repetitive practice that XR provides augments non-virtual (traditional) simulation training. |
| There are assumptions and ideas built into the statements with which I do not always agree, confounding the rating for me. If I disagree with a statement, I am tempted to rate it lower in importance. Which muddies the waters. Example: E.g. the second item XR Will be more available. TOTALLY untrue if the setting lacks electricity or internet or weak internet. |
| Suggestion for wording of Research and Development item above - separate out ethical concerns from logistics and from the possibility of new ways to study biological processes - these are all independent items in my view and I don’t ever see the possibility of R&D without ethical concerns |
| Research and Development: XR can be used to study complex human behaviors in a simulated setting, including studying human physiology changes for stress while in XR settings alone or in a team. |
| Regional variations may be one of the biggest barriers to widespread adoption. Similarly, overly standardized training may encounter problems if supply chain or specific equipment availability limits applicability of programmed education. |
| In my experience, patient engagement is a very good idea, but most of the patient population I know might be overwhelmed and scared by the technology. Also, access to education in ressource-scarce areas, basing it on high-level technology, is kind of a contradiction in itself. These devices need power, internet connection, etc. |
| Cultural sensitivity - XR programs should include culturally relevant scenarios that reflect the unique health challenges, languages, and traditions of rural and Indigenous communities. This ensures that training is not only technically sound but also socially and culturally appropriate for rural practitioners. |
| Assessment of competence: XR can be used to assess knowledge, skills, and attitudes in a highly standardized and patient- safe environment - thereby ensuring that all trainees achieve necessary competence before proceeding to actual patient encounters. |

Barriers Identified

Technological Limitations

**Infrastructure:** Lack of necessary hardware and software infrastructure can hinder the adoption of XR. This includes insufficient computing power, lack of VR-ready devices, and inadequate network capabilities. **73%**

| 1 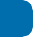 | 5% |  |  |  |  |  | Number of answers  1 |
| --- | --- | --- | --- | --- | --- | --- | --- |
| 2 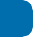 | 5% |  |  |  |  |  | 1 |
| 3 | 18% |  |  |  |  |  | 4 |
| 4 |  | 32% |  |  |  |  | 7 |
| 5 |  |  | 41% |  |  |  | 9 |
| 0% |  | 25% |  | 50% | 75% | 100% |  |

**Usability Issues:** Complexities in using XR systems, such as user-unfriendly interfaces or cumbersome equipment, can deter faculty and students from fully engaging with the technology. **77%**

Number of answers

1 0

0%

0%

23%

32%

45%

2 0

3 5

4 7

5 10

0% 25% 50% 75% 100%

**Cybersickness and Physical Discomfort**: Symptoms like nausea, eye strain, and disorientation while using XR can limit prolonged use and negatively affect learning experiences. **37%**

| 1 | 0% |  |  |  |  | Number of answers  0 |
| --- | --- | --- | --- | --- | --- | --- |
| 2 | 14% |  |  |  |  | 3 |
| 3 |  |  | 50% |  |  | 11 |
| 4 |  | 32% |  |  |  | 7 |
| 5 | 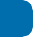 5% |  |  |  |  | 1 |
|  | 0% | 25% | 50% | 75% | 100% |  |

**Financial Constraints - High Initial costs**

**Infrastructure and Hardware:** Upgrading existing IT infrastructure to support the data and graphical requirements of XR technologies often requires significant investment. XR hardware, including VR headsets, AR glasses, and associated peripherals, can be expensive, especially when purchasing enough units to support large classes. **68%**

| 1 | 0% |  |  |  |  | Number of answers  0 |
| --- | --- | --- | --- | --- | --- | --- |
| 2 | 0% |  |  |  |  | 0 |
| 3 |  | 32% |  |  |  | 7 |
| 4 |  | 32% |  |  |  | 7 |
| 5 |  | 36% |  |  |  | 8 |
|  | 0% | 25% | 50% | 75% | 100% |  |

**Software and Updates:** Licensing fees for high-quality XR software or custom development costs for specific educational applications can be substantial. Continuous software updates are necessary to keep XR applications functional and secure, which can incur ongoing costs. **82%**

Number of answers

1 0


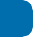


0%

5%

14%

55%

27%

2 1

3 3

4 12

5 6

0% 25% 50% 75% 100%

Scaling Challenges

**Expansion Costs:** Scaling XR implementations from pilot projects to full integration across multiple courses or departments can be financially demanding. **78%**

Number of answers

1 0


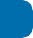


0%

5%

18%

64%

14%

2 1

3 4

4 14

5 3

0% 25% 50% 75% 100%

**Sustainability:** Ensuring the long-term sustainability of XR initiatives, including funding for ongoing support staff and technology refresh cycles, can strain budgets. **73%**

| 1 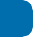 | 5% |  |  |  |  | Number of answers  1 |
| --- | --- | --- | --- | --- | --- | --- |
| 2 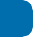 | 5% |  |  |  |  | 1 |
| 3 | 18% |  |  |  |  | 4 |
| 4 |  |  | 50% |  |  | 11 |
| 5 | 23% |  |  |  |  | 5 |
| 0% |  | 25% | 50% | 75% | 100% |  |

**Cultural and Organizational Resistance**

**Resistance to Change:** Faculty and administrative resistance to integrating new technologies into traditional curricula can be a significant barrier. **63%**

| 1 | 0% |  |  |  |  | Number of answers  0 |
| --- | --- | --- | --- | --- | --- | --- |
| 2 |  | 23% |  |  |  | 5 |
| 3 | 14% |  |  |  |  | 3 |
| 4 |  |  | 36% |  |  | 8 |
| 5 |  | 27% |  |  |  | 6 |

0% 25% 50% 75% 100%

Lack of Awareness and Understanding: Insufficient knowledge about the benefits and potential applications of XR in education can result in underutilization. **82%**

| 1 | 0% |  |  |  |  |  | Number of answers  0 |
| --- | --- | --- | --- | --- | --- | --- | --- |
| 2 | 9% |  |  |  |  |  | 2 |
| 3 |  | 18% |  |  |  |  | 4 |
| 4 |  |  | 27% |  |  |  | 6 |
| 5 |  |  |  | 45% |  |  | 10 |

0% 25% 50% 75% 100%

**Regulatory and Ethical Issues**

**Privacy Concerns:** Handling sensitive data within XR environments poses privacy risks that must be addressed through strict data management protocols. **50%**

| 1 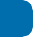 | 5% |  |  |  |  |  | Number of answers  1 |
| --- | --- | --- | --- | --- | --- | --- | --- |
| 2 |  | 23% |  |  |  |  | 5 |
| 3 |  | 23% |  |  |  |  | 5 |
| 4 |  |  | 36% |  |  |  | 8 |
| 5 | 14% |  |  |  |  |  | 3 |
| 0% |  |  | 25% | 50% | 75% | 100% |  |

**Ethical Use:** Ethical dilemmas, such as the potential for bias in virtual scenarios or the psychological impact of immersive simulations, need careful consideration. **32%**

| 1 | 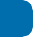 | 5% |  |  |  |  | Number of answers  1 |
| --- | --- | --- | --- | --- | --- | --- | --- |
| 2 |  |  | 27% |  |  |  | 6 |
| 3 |  |  |  | 36% |  |  | 8 |
| 4 |  |  | 23% |  |  |  | 5 |
| 5 | 9% |  |  |  |  |  | 2 |

0% 25% 50% 75% 100%

| **Comments/Suggestions for Other Topic Areas not Listed Above:** |
| --- |
| subscription-based programming and portions of software control/upgrades being outside of the program's control make consistency of adoption a concern. Tethered devices to minimize impact of battery requirements are becoming less common. |
| This part of the survey addresssed the issues I raised in the first part. Thanks. |
| The main priority should be surrounding comprehensive business plan for implementation of new technology which should be developed based on a comprehensive needs assessment and key partnership (stakeholder) buy in at every stage.  Further, this process should be developed with a framework that supports change (ie Kotter's Change Theory 1996) with the elements of a digital transformation that has been adopted by the business industry.  Having a plan for cyber sickness or alternative methods of education is a must. Assume that not every user can use the technology and have a back up plan (ADA compliance, closed captioning, visual casting...) |
| Limited scientific evidence for the efficacy of XR training is a major barrier to widespread implementation. |
| Lack or awareness and resistance to change are important - but these cannot be overcome until the technology is advanced enough and more cost effective. So, for me, the latter is a higher priority so that we can then address the former (awareness & resistance).  As for the regulatory and ethical issues, these are critical...but there already exist many frameworks and protocols that can be extended to this area. |
| Cybersickness will become less and less of an issue, as more natural movement patterns evolve in VR (no teleporting). However, devices bound to certain providers (Meta comes to mind) might pose a huge challenge to different organisations. |
| Cultural and Contextual Appropriateness of XR Content: XR programs developed for urban healthcare settings may not be fully applicable to rural contexts, where healthcare delivery can be vastly different. More emphasis should be placed on tailoring XR content to the specific realities of rural practice, such as resource scarcity and long distances to tertiary care centers. |

Facilitators Identified Strategic Partnerships

**Collaborations with Technology Providers:** Partnering with XR hardware and software vendors can facilitate access to cutting-edge technology and technical support. **87%**

| 1 | 0% |  |  |  |  | Number of answers  0 |
| --- | --- | --- | --- | --- | --- | --- |
| 2 | 0% |  |  |  |  | 0 |
| 3 | 14% |  |  |  |  | 3 |
| 4 |  |  |  | 64% |  | 14 |
| 5 |  | 23% |  |  |  | 5 |

0% 25% 50% 75% 100%

**Academic and Industry Partnerships:** Collaborations can provide mutual benefits, including shared resources, expertise, and funding opportunities. **96%**

Number of answers

1 0


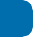


0%

0%

5%

41%

55%

2 0

3 1

4 9

5 12

0% 25% 50% 75% 100%

**Policy and Framework Development**

**Institutional Policies:** Developing clear policies that support the adoption and ethical use of XR technologies can provide a structured framework for integration. **63%**

| 1 | 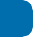 | 5% |  |  |  |  |  | Number of answers  1 |
| --- | --- | --- | --- | --- | --- | --- | --- | --- |
| 2 | 9% |  |  |  |  |  |  | 2 |
| 3 |  |  | 23% |  |  |  |  | 5 |
| 4 |  |  |  | 36% |  |  |  | 8 |
| 5 |  |  | 27% |  |  |  |  | 6 |

0% 25% 50% 75% 100%

**Curricular Integration Guidelines:** Guidelines that help faculty incorporate XR into the curriculum systematically can facilitate smoother adoption. **73%**

Number of answers

1 0

0%

9%

18%

41%

32%

2 2

3 4

4 9

5 7

0% 25% 50% 75% 100%

**Evidence-based Practice**

Research and Case Studies: Demonstrating the effectiveness of XR through research and successful case studies can help overcome skepticism and build a case for wider adoption. **82%**

Number of answers

1 0


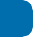


0%

5%

14%

23%

59%

2 1

3 3

4 5

5 13

0% 25% 50% 75% 100%

**Outcome Measures**: Robust metrics to evaluate the impact of XR on learning outcomes can justify investment and encourage broader use. **91%**

Number of answers

1 0

0%

0%

9%

36%

55%

2 0

3 2

4 8

5 12

0% 25% 50% 75% 100%

| **Comments/Suggestions for Other Topic Areas not Listed Above:** |
| --- |
| Under evidence based practice - need to demonstrate impact directly on care / healthcare professionals not just learning outcomes. The holy grail for outcomes is when patient care improves or there’s a direct impact on health Human Resources. |
| Partnership are critical for either "home grown" technology where one partners across an academic center to collaborate and develop and implement new technology or if working with a vendor for "off the shelf" technology. The goal must center around the curricular needs and the learning objectives established by the faculty/program and not the vendor. |
| I believe really, concretely measuring learning outcomes, not only in the learning environment but also in real world, could be a strong argument in favour of XR |
| Establish regional collaborative networks among healthcare providers, educators, and institutions. These networks could facilitate knowledge sharing, exchange of best practices, and joint funding applications for XR technology adoption, creating a sense of community ownership and support for XR initiatives. |
| Differentiation and pedagogic understanding of implementation use cases for skill vs cognitive interactions with trustable skill/knowledge feedback will be required for larger-scale use. |

**Area of Need Technological Proficiency**

**Training:** Faculty members need comprehensive training on the technical aspects of XR technologies. This includes understanding the hardware and software, troubleshooting common issues, and staying updated on the latest developments. **59%**

| 1 | 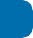 | 5% |  |  |  |  | Number of answers  1 |
| --- | --- | --- | --- | --- | --- | --- | --- |
| 2 | 9% |  |  |  |  |  | 2 |
| 3 |  |  | 27% |  |  |  | 6 |
| 4 |  | 18% |  |  |  |  | 4 |
| 5 |  |  |  | 41% |  |  | 9 |

0% 25% 50% 75% 100%

**Skill Development:** Developing skills for effectively integrating XR tools into the educational process. This includes scenario design, instructional design tailored to XR, and adapting teaching styles to immersive learning environments. **87%**

Number of answers

1 0


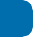


0%

5%

9%

32%

55%

2 1

3 2

4 7

5 12

0% 25% 50% 75% 100%

Pedagogical Integration

**Curriculum Development:** Designing curricula that incorporate XR in a way that enhances learning outcomes. Faculty should understand how to use XR to complement traditional teaching methods rather than replace them.**91%**

| 1 | 0% |  |  |  |  | Number of answers  0 |
| --- | --- | --- | --- | --- | --- | --- |
| 2 | 0% |  |  |  |  | 0 |
| 3 | 9% |  |  |  |  | 2 |
| 4 |  | 32% |  |  |  | 7 |
| 5 |  |  | 59% |  |  | 13 |
|  | 0% | 25% | 50% | 75% | 100% |  |

**Instructional Design:** Training on how to create engaging and educationally effective XR experiences. Developing competencies and teaching methodologies that leverage XR technologies effectively (scenario-based learning, gamification, interactive design principles). **86%**

|  | | | | | Number of  answers |
| --- | --- | --- | --- | --- | --- |
| 1 | 0% |  |  |  | 0 |
| 2 | 0% |  |  |  | 0 |
| 3 | 14% |  |  |  | 3 |
| 4 |  | 41% |  |  | 9 |
| 5 |  | 45% |  |  | 10 |

0% 25% 50% 75% 100%

Assessment and Evaluation

**Outcome Measures:** Educating faculty on how to measure and evaluate the impact of XR on learning outcomes. This includes understanding which metrics are most relevant for XR-based education and how to collect and analyze this data. **91%**

| 1 | 0% |  |  |  |  | Number of answers  0 |
| --- | --- | --- | --- | --- | --- | --- |
| 2 | 0% |  |  |  |  | 0 |
| 3 | 9% |  |  |  |  | 2 |
| 4 |  | 32% |  |  |  | 7 |
| 5 |  |  | 59% |  |  | 13 |
|  | 0% | 25% | 50% | 75% | 100% |  |

**Continuous Improvement:** Training faculty to use feedback and data from XR sessions to refine & improve the educational content & delivery 77%

| 1 | 0% |  |  |  |  | Number of answers  0 |
| --- | --- | --- | --- | --- | --- | --- |
| 2 | 0% |  |  |  |  | 0 |
| 3 |  | 23% |  |  |  | 5 |
| 4 |  |  | 50% |  |  | 11 |
| 5 |  | 27% |  |  |  | 6 |
|  | 0% | 25% | 50% | 75% | 100% |  |

**Ethical and Practical Considerations**

**Accessibility and Inclusivity:** Ensuring faculty are aware of and know how to address potential accessibility issues with XR, making the technology inclusive for all students. **77%**

| 1 | 0% |  |  |  |  | Number of answers  0 |
| --- | --- | --- | --- | --- | --- | --- |
| 2 | 9% |  |  |  |  | 2 |
| 3 | 14% |  |  |  |  | 3 |
| 4 |  | 36% |  |  |  | 8 |
| 5 |  | 41% |  |  |  | 9 |
|  | 0% | 25% | 50% | 75% | 100% |  |

**Ethical Use of Technology:** Understanding the ethical implications of using immersive technology in education, including privacy concerns and the psychological impact on students. **68%**

| 1 | 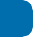 | 5% |  |  |  |  |  | Number of answers  1 |
| --- | --- | --- | --- | --- | --- | --- | --- | --- |
| 2 | 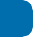 | 5% |  |  |  |  |  | 1 |
| 3 |  |  | 23% |  |  |  |  | 5 |
| 4 |  |  |  | 41% |  |  |  | 9 |
| 5 |  |  | 27% |  |  |  |  | 6 |
|  | 0% |  | 25% |  | 50% | 75% | 100% |  |

| **Comments/Suggestions for Other Topic Areas not Listed Above:** |
| --- |
| This area is the key to success. A well established plan for adoption and implementation is critical. The simulation educators need to stress the importance of simulation standards of best practice regardless of the modality. Technology usage for training when recreating a healthcare environment or covering elements that are addressed in simulation-based education should be viewed as a another modality and simulation pedagogy should drive the process, including based on a learning theory, pilot testing, faculty training, pre-briefing, debriefing, and evaluation. |
| In addition to making simulation inclusive for all learners, it will also be important to attend to any systematic biases & inequities that might be perpetuated in a virtual environment. |
| Faculty need to understand how impact goes beyond learning outcomes and directly impacts care. Also important for faculty to understand impact on the learner for items not directly related to learning objectives - eg. PTSD. |
| Consider the additional workload of learning and integrating XR technologies, as faculty often juggle multiple responsibilities. Developing strategies to manage and balance these demands (e.g., workload adjustments, dedicated XR training time) would be key to supporting faculty in successfully adopting and implementing XR in their teaching practices. |

Area of Focus Effectiveness and Impact

Develop and validate measures to assess the effectiveness and impact of XR applications. **91%**

| 1 | 0% |  |  |  |  | Number of answers  0 |
| --- | --- | --- | --- | --- | --- | --- |
| 2 | 0% |  |  |  |  | 0 |
| 3 | 9% |  |  |  |  | 2 |
| 4 |  | 36% |  |  |  | 8 |
| 5 |  |  | 55% |  |  | 12 |
|  | 0% | 25% | 50% | 75% | 100% |  |

Comparative studies to establish the efficacy of different XR modalities. **72%**

| 1 | 0% |  |  |  |  |  | Number of answers  0 |
| --- | --- | --- | --- | --- | --- | --- | --- |
| 2 |  | 18% |  |  |  |  | 4 |
| 3 | 9% |  |  |  |  |  | 2 |
| 4 |  |  | 36% |  |  |  | 8 |
| 5 |  |  | 36% |  |  |  | 8 |
|  | 0% |  | 25% | 50% | 75% | 100% |  |

Outcome Measures

Validation of study outcomes and effectiveness of XR in changing clinical practice and behavior. **91%**

Number of answers

1 0


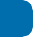

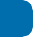


0%

5%

5%

32%

59%

2 1

3 1

4 7

5 13

0% 25% 50% 75% 100%

Technological Challenges

Continued development and refinement of XR technologies to address identified user challenges and limitations. **68%**

Number of answers

1 0


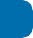


0%

5%

27%

36%

32%

2 1

3 6

4 8

5 7

0% 25% 50% 75% 100%

Translational Research Outcomes

Create actionable frameworks & guidelines for the sustainable implementation of XR in healthcare settings, especially in underserved regions. **81%**

| 1 | 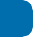 | 5% |  |  |  |  |  | Number of answers  1 |
| --- | --- | --- | --- | --- | --- | --- | --- | --- |
| 2 | 9% |  |  |  |  |  |  | 2 |
| 3 | 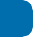 | 5% |  |  |  |  |  | 1 |
| 4 |  |  |  | 45% |  |  |  | 10 |
| 5 |  |  | 36% |  |  |  |  | 8 |
|  | 0% |  | 25% |  | 50% | 75% | 100% |  |

Methodological Rigor and Validation

**Extent of valid use of extended reality applications.** Determining the extent of valid use involves identifying the specific educational settings and scenarios where XR provides significant learning benefits, which is currently underexplored and lacks comprehensive guidelines. **81%**

| 1 | 0% |  |  |  |  |  |  | Number of answers  0 |
| --- | --- | --- | --- | --- | --- | --- | --- | --- |
| 2 | 0% |  |  |  |  |  |  | 0 |
| 3 |  | 18% |  |  |  |  |  | 4 |
| 4 |  |  | 36% |  |  |  |  | 8 |
| 5 |  |  |  | 45% |  |  |  | 10 |
|  | 0% |  | 25% |  | 50% | 75% | 100% |  |

**Completion of full validation processes for technologies.** There is a gap in fully validating XR technologies through all stages of development and deployment, ensuring that they meet educational objectives and maintain effectiveness over time. **72%**

| 1 | 0% |  |  |  |  |  |  | Number of answers  0 |
| --- | --- | --- | --- | --- | --- | --- | --- | --- |
| 2 | 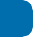 | 5% |  |  |  |  |  | 1 |
| 3 |  |  | 23% |  |  |  |  | 5 |
| 4 |  |  |  | 36% |  |  |  | 8 |
| 5 |  |  |  | 36% |  |  |  | 8 |
|  | 0% |  |  | 25% | 50% | 75% | 100% |  |

**Standardization and comparability of study designs.** The absence of standardized study designs in XR research leads to challenges in comparing results across different studies, hindering the synthesis of evidence and the development of generalizable conclusions. **77%**

| 1 | 0% |  |  |  |  |  | Number of answers  0 |
| --- | --- | --- | --- | --- | --- | --- | --- |
| 2 | 0% |  |  |  |  |  | 0 |
| 3 |  | 23% |  |  |  |  | 5 |
| 4 |  |  | 41% |  |  |  | 9 |
| 5 |  |  | 36% |  |  |  | 8 |
|  | 0% |  | 25% | 50% | 75% | 100% |  |

**Need for higher-quality studies and robust evidence.** There is a critical need for higher-quality research studies that employ rigorous methodologies and robust evidence collection to substantiate the claims made about the educational impacts of XR technologies. **100%**

| 1 | 0% |  |  |  |  |  | Number of answers  0 |
| --- | --- | --- | --- | --- | --- | --- | --- |
| 2 | 0% |  |  |  |  |  | 0 |
| 3 | 0% |  |  |  |  |  | 0 |
| 4 |  |  | 41% |  |  |  | 9 |
| 5 |  |  |  | 59% |  |  | 13 |
|  | 0% | 25% |  | 50% | 75% | 100% |  |

**Lack of validity in Study conclusions.** Many studies on XR applications suffer from validity issues in their conclusions, often due to methodological weaknesses, inadequate controls, or biases in data interpretation, which compromises their reliability and applicability. **91%**

| 1 | 0% |  |  |  |  | Number of answers  0 |
| --- | --- | --- | --- | --- | --- | --- |
| 2 | 0% |  |  |  |  | 0 |
| 3 | 9% |  |  |  |  | 2 |
| 4 |  |  | 55% |  |  | 12 |
| 5 |  | 36% |  |  |  | 8 |
|  | 0% | 25% | 50% | 75% | 100% |  |

| **Comments/Suggestions for Other Topic Areas not Listed Above:** |
| --- |
| There already exist measures and guidelines for assessing the effectiveness and impact of technological innovations and educational programs. Many of these could readily be applied to this domain.  In addition to overcoming the technological and cost issues, determining the extent of valid use of extended reality applications is a key endeavor for our field. It is not - and will never be - the best educational modality for many skills, and it will be important to determine where XR will really bring an advantage...and concentrate usage in those areas (given that the costs of this technology are likely to stay elevated for a long time) |
| Studies should aim at exploring outcomes on higher level of Kirkpatrick's pyramid. Studies only exploring "Reactions" (e.g. the trainees liked the experience) are not good enough.  Studies should not compare something (XR) to nothing. |
| I believe the technological challenges can and will be addressed more on the part of the technology companies, here we as researchers in the educational field can only provide feedback through established partnerships.  Methodological rigour and validation are key aspects, but sadly not only in the study of XR, but in the study of simulation in general - so I'm not sure XR needs a special emphasis on these aspects |
| Conduct comprehensive cost-benefit analyses to determine the financial viability of implementing XR in healthcare education. This research could help institutions weigh the initial costs of XR adoption against long-term benefits, such as improved learning outcomes and reduced need for physical simulation equipment. |
| Adoption of a standardized approach to technology integration and based on standards of best practice and a shared language around terminology will help with the research process. However, there is a lack of validated tools, and reporting guidelines surrounding the research of various XR technologies. Like most simulation-based education in healthcare, more rigor and higher level of research needs to be established. |
| Additional gaps that may be considered:   - A lack of longitudinal and large-scale studies to assess the long-term effectiveness and impact of XR applications. - An overemphasis on technical skills training, with insufficient exploration of broader clinical competencies. - A predominant focus on education and training for healthcare professionals, rather than expanding XR applications to patient-centered care (e.g., personalized treatment) or system-level approaches (e.g., healthcare system optimization) |
