# Supplementary material for: Setting a research agenda for the use of extended reality in healthcare simulation: an Utstein style meeting
Source: Adv Simul (Lond). 2026 Mar 3;11:16. doi: 10.1186/s41077-026-00409-y (PMC12954913; doi:10.1186/s41077-026-00409-y)
Supplement: Supplementary file 4 — Supplementary Material 4. [file 41077_2026_409_MOESM4_ESM.docx]

Utstein Meeting on the Use of Extended Reality in Healthcare Simulation Agenda

Day 1: 23 November 2024

| **Begin** | **End** | **Session Name** | **Session Type** |
| --- | --- | --- | --- |
| 09:00 | 09:30 | Welcome and introduction to the program and the Utstein Meetings  Doris Østergaard | Plenum |
| 09:30 | 09:45 | Global Concesus Statement on Simulation-based Practice in Healthcare & working definitions for Extended Reality  Francisco Matos & Pier Luigi Ingrassia | Plenum |
| 09:45 | 10:15 | Short overview responses to survey for Goal 1  Barry Issenberg | Plenum |
| 10:15 | 10:35 | Coffee and find your group |  |
| 10:35 | 11:30 | Small group session 1 - Identify, review and discuss uses of XR in  simulation-based education | All groups |
| 11:30 | 12:00 | Presentation of group work | Plenum |
| 12:00 | 13:00 | Lunch |  |
| 13:00 | 13:20 | Review morning session and goals for afternoon session - short review of survey responses for Goals 2a & 2b  Barry Issenberg | Plenum |
| 13:20 | 13:55 | Small group session 2: Grps 1 &3: Goal 2a - Review and discuss challenges and barriers to using XR in SBE; Grps 2&4:  Facilitators/solution to using XR in SBE | Groups |
| 13:55 | 14:30 | Small group sessions: Groups 1&3 switch with 2&4 | Groups |
| 14:30 | 15:15 | Presentation of group work | Plenum |
| 15:15 | 15:30 | Coffee |  |
| 15:30 | 15:45 | Presentation for goals of next session - review survey responses for Goal 3 - faculty development  Barry Issenberg | Plenum |
| 15:45 | 16:45 | Small group session 3 | Groups |
| 16:45 | 17:15 | Presentation of group work | Plenum |
| 17:15 | 17:30 | Wrap up and plan for Day 2  Barry Issenberg and Doris Østergaard | Plenum |

Day 2: 24 November 2024

| **Begin** | **End** | **Session Name** | **Session Type** |
| --- | --- | --- | --- |
| 09:00 | 09:30 | Review day 1 and what should be obtained today  Francisco Matos and Barry Issenberg | Plenum |
| 09:30 | 09:45 | Brief for 1st Session - Review responses from survey  Barry Issenberg | Plenum |
| 09:45 | 10:30 | Small group session | Plenum |
| 10:30 | 10:45 | Coffee |  |
| 10:45 | 11:30 | Small group session – continue | Groups |
| 11:00 | 12:00 | Presentation of group work | Plenum |
| 12:00 | 13:00 | Lunch |  |
| 13:00 | 13:15 | Review morning session and goals for afternoon session | Plenum |
| 13:15 | 14:00 | Small group session | Groups |
| 14:00 | 14:30 | Presentation of group work | Plenum |
| 14:30 | 14:45 | The next steps | Plenum |
| 14:45 | 15:00 | Evaluation of the meeting | Plenum |
